# Beyond screen time: commentary on early screen media multitasking as a developmental risk for executive function in preschoolers

**DOI:** 10.1038/s41390-025-04341-1

**Published:** 2025-08-21

**Authors:** Xiaopeng Ji, Naixue Cui, Jianghong Liu

**Affiliations:** 1 School of Nursing, College of Health Sciences, University of Delaware, Newark, DE USA; 2 School of Nursing and Rehabilitation, Cheeloo College of Medicine, Shandong University, Jinan, Shandong China; 3 Department of Family and Community Health, School of Nursing, University of Pennsylvania, Philadelphia, PA USA

The widespread use of digital media in early childhood raises important concerns for cognitive and behavioral development.^1,2^ The American Academy of Pediatrics recommends limiting screen time and promoting parent-child shared media use to support education and learning in young children under five.^3^ Moving beyond screen time, Srisinghasongkram and colleagues examined the predictive effect of screen media multitasking (SMM), defined as the concurrent use of two or more screens, on executive function in young children.^1^ Executive function is an interrelated set of higher-order cognitive skills, such as inhibitory control, working memory, attention, and cognitive flexibility. Executive function, key to behavior, emotions, and social skills, develops rapidly in the first five years.^4,5^ Study findings from Srisinghasongkram and colleagues offer valuable insights into the complex risk profile of SMM, underscoring the need for more targeted guidance for parents and caregivers.

SMM, such as watching TV while using a smartphone or tablet, has become increasingly common among children, with behavioral adoption beginning as early as infancy. Srisinghasongkram and colleagues utilized a prospective cohort design tracking 280 children in Thailand from 6 months to 4 years of age,^1^ which is a notable strength that permits examination of long-term trajectories. SMM exposure was reported in 6.9% of infants at 6 months, rising to 28% by 18 months and 36.8% by age 4. Research shows similar trend in the United States, where nearly all children under one use mobile devices, and about one-third of preschoolers engage in SMM.^6^ Similar findings across nations highlight SMM as a relatively new media use pattern emerging globally in early childhood, necessitating urgent attention from researchers, policymakers, and parents. However, in Srisinghasongkram’s study, the use of 24-h media diary on a weekday may introduce bias due to recall bias and the omission of typically different weekend media habits.^7^

Srisinghasongkram and colleagues extended prior research by identifying differential impacts between daily SMM duration, prolonged exposure periods, and frequency of repeated exposure.^1^ Most previous research has examined cognitive outcomes associated with single media use^8^ or focused on SMM use in adolescents and adults.^9^ Compared to peers with single or less frequent media use, adolescents with higher social media multitasking show poorer academics, reduced attention and working memory, greater impulsivity, and difficulty with deep processing.^9,10^ Early childhood represents a period of remarkable neuroplasticity, making it a critical period for understanding the impact of screen media on neurocognitive development. A meta-analysis has linked excessive single media use to shorter sleep duration, impaired executive function, delayed motor skills, and increased aggression in toddlers and preschoolers.^11^ Srisinghasongkram’s study further suggests that prolonged and repeated SMM exposure exerted more significant negative influence on executive function and general cognitive ability compared to other exposure patterns. While the multidimensional analytical approach offers a more nuanced risk profile, the study’s reliance on assumed consistency in media use patterns over time represents a methodological limitation that may reduce measurement accuracy.

The longitudinal study cited did not specifically explore differences among executive function domains in relation to SMM exposure.^1^ Compared to single media engagement, SMM imposes a greater cognitive load due to the demands of attention division and frequent task-switching.^12,13^ This constant reallocation of cognitive resources may overwhelm children’s developing executive function during early childhood.^12^ Therefore, individuals with high SMM use tend to have difficulties with tasks that require sustained, goal-directed attention.^13^ Although the majority of evidence points to either detrimental effects or no significant association between SMM and executive function,^14^ some studies suggest that high SMM levels may function as a form of cognitive training, potentially enhancing task-switching ability.^15^ Future research should investigate how specific executive function domains are affected by SMM exposure.

Moreover, the impact of SMM on executive function may depend on the timing of screen media use and specific media characteristics. Moderate evening screen time (after dinner, before bedtime) under 2 h shows minimal negative effects. However, any night-time screen use (at bedtime or after lights out) is linked to sleep deprivation, insomnia risk, and daytime sleepiness.^16^ These sleep disruptions directly impair executive function.^17^ Media characteristics, such as presentation pace, educational value and interactive feature, are also significant predictors of cognitive development in children.^18,19^ For example, moderate use of high-quality, developmentally appropriate educational programs, such as “Sesame Street”, can enhance executive function compared to non-educational content.^20^ Additionally, children with high exposure to passive media such as television, video and social media are associated with lower cognitive function.^21^ In contrast, those who engage with interactive media, particularly action or educational video games, often show better cognitive performance, probably due to the beneficial effect on top-down cognitive processes.^8,21^ However, the longitudinal study cited did not differentiate between media use timing, specific content types or media formats, highlighting a crucial gap in our understanding.^1^ Future research that examines children’s media use patterns, accounting for timing, content type, media format, and their distinct cognitive impacts, would provide valuable insights for developing evidence-based recommendations for parents and caregivers.

Parental involvement critically shapes both children’s media use patterns and executive function development. Srisinghasongkram’s longitudinal study replicated previous findings by showing that positive parenting and mother–child interaction correlate with decreased SMM exposure, as well as enhanced cognitive development and better executive function at age 3.^1^ Considering the current evidence, parent involvement and parent-child interaction may play crucial roles, such as mediators, moderators, or confounding variables, in the relationship between SMM and executive function. However, this study does not sufficiently examine these specific roles of parental influence, which limits the practical applications of these findings. Caregivers often report using screen media primarily as a “digital pacifier” to manage behaviors, support sleep routines, and allow time for household responsibilities,^6^ suggesting pragmatic parenting solutions that may drive problematic media habits during this critical developmental window. Future studies should incorporate paternal influences, diverse family structures beyond mother-child dyads, and cultural variations in SMM behaviors to develop more comprehensive models of how caregiver dynamics influence SMM-related disruptions to executive function.

In summary, screen media multitasking is an emerging pattern of screen use beginning in infancy and early childhood.^22^ Early screen experiences shape lasting media habits and may significantly influence executive function development, particularly during the critical period before age 5. Evidence-based guidance should move beyond simple screen time limits to address more nuanced factors such as engagement patterns (e.g., multi-screen use, timing of use) and content quality. Future research should examine how specific executive function domains are affected by SMM exposure, considering factors such as dose-response effects, timing of screen media use, content types, media format, age-appropriate use and influences of parents or caregivers. A more comprehensive approach can better support parents, educators, and policymakers in fostering healthy media habits that align with children’s cognitive and developmental needs.

## References

[1] Srisinghasongkram, P., Trairatvorakul, P., Maes, M. & Chonchaiya, W. Early screen media multitasking associated with executive function problems in preschool-age children. *Pediatr. Res*. 1–11 (2025) 10.1038/s41390-025-04053-6. 10.1038/s41390-025-04053-6 40210956

[2] Zhao, J. et al. Relationship between screen use and internalizing/externalizing problems among preschoolers: the mediation of circadian rhythm. *Pediatr. Res*. 1–8 (2025) 10.1038/s41390-025-03944-y. 10.1038/s41390-025-03944-y 39987340

[3] Hill, D. et al. Media and young minds. *Pediatrics* **138**, e20162591 (2016). 10.1542/peds.2016-2591 27940793

[4] Anderson, P. Assessment and development of executive function (EF) during childhood. *Child Neuropsychol.* **8**, 71–82 (2002). 12638061 10.1076/chin.8.2.71.8724

[5] Garon, N., Bryson, S. E. & Smith, I. M. Executive function in preschoolers: a review using an integrative framework. *Psychol. Bull.* **134**, 31 (2008). 18193994 10.1037/0033-2909.134.1.31

[6] Kabali, H. K. et al. Exposure and use of mobile media devices by young children. *Pediatrics* **136**, 1044–1050 (2015). 26527548 10.1542/peds.2015-2151

[7] Vandewater, E. A. & Lee, S.-J. Measuring children’s media use in the digital age: issues and challenges. *Am. Behav. Sci.* **52**, 1152–1176 (2009). 19763246 10.1177/0002764209331539 PMC2745155

[8] Zhang, Z. et al. Associations between screen time and cognitive development in preschoolers. *Paediatr. Child Health* **27**, 105–110 (2022). 35599677 10.1093/pch/pxab067 PMC9113848

[9] Van Der Schuur, W. A., Baumgartner, S. E., Sumter, S. R. & Valkenburg, P. M. The consequences of media multitasking for youth: a review. *Comput. Hum. Behav.* **53**, 204–215 (2015).

[10] Cain, M. S., Leonard, J. A., Gabrieli, J. D. & Finn, A. S. Media multitasking in adolescence. *Psychonomic Bull. Rev.* **23**, 1932–1941 (2016). 10.3758/s13423-016-1036-3 27188785

[11] Li, C., Cheng, G., Sha, T., Cheng, W. & Yan, Y. The relationships between screen use and health indicators among infants, toddlers, and preschoolers: a meta-analysis and systematic review. *Int. J. Environ. Res. Public Health* **17**, 7324 (2020). 33036443 10.3390/ijerph17197324 PMC7579161

[12] Courage, M. L., Bakhtiar, A., Fitzpatrick, C., Kenny, S. & Brandeau, K. Growing up multitasking: the costs and benefits for cognitive development. *Dev. Rev.* **35**, 5–41 (2015).

[13] Ralph, B. C., Thomson, D. R., Seli, P., Carriere, J. S. & Smilek, D. Media multitasking and behavioral measures of sustained attention. *Atten. Percept. Psychophys.* **77**, 390–401 (2015). 25280520 10.3758/s13414-014-0771-7

[14] Uncapher, M. R. et al. Media multitasking and cognitive, psychological, neural, and learning differences. *Pediatrics* **140**, S62–S66 (2017). 29093034 10.1542/peds.2016-1758D PMC5658797

[15] Alzahabi, R. & Becker, M. W. The association between media multitasking, task-switching, and dual-task performance. *J. Exp. Psychol. Hum. Percept. Perform.* **39**, 1485 (2013). 23398256 10.1037/a0031208

[16] Hartley, S., Royant-Parola, S., Zayoud, A., Gremy, I. & Matulonga, B. Do both timing and duration of screen use affect sleep patterns in adolescents? *Plos One* **17**, e0276226 (2022). 36264928 10.1371/journal.pone.0276226 PMC9584513

[17] Liu, J. et al. Childhood sleep: physical, cognitive, and behavioral consequences and implications. *World J. Pediatr.* **20**, 122–132 (2024). 36418660 10.1007/s12519-022-00647-w PMC9685105

[18] Rhodes, S. M., Stewart, T. M. & Kanevski, M. Immediate impact of fantastical television content on children’s executive functions. *Br. J. Dev. Psychol.* **38**, 268–288 (2020). 31872905 10.1111/bjdp.12318

[19] Lillard, A. S. & Peterson, J. The immediate impact of different types of television on young children’s executive function. *Pediatrics* **128**, 644–649 (2011). 21911349 10.1542/peds.2010-1919 PMC9923845

[20] Anderson, D. R., Subrahmanyam, K. & Workgroup CIoDM. Digital screen media and cognitive development. *Pediatrics* **140**, S57–S61 (2017). 29093033 10.1542/peds.2016-1758C

[21] Walsh, J. J., Barnes, J. D., Tremblay, M. S. & Chaput, J.-P. Associations between duration and type of electronic screen use and cognition in US children. *Comput. Hum. Behav.* **108**, 106312 (2020).

[22] Ponti, M. Screen time and preschool children: promoting health and development in a digital world. *Paediatr. Child Health* **28**, 184–192 (2023). 37205134 10.1093/pch/pxac125 PMC10186096

